# Interpreting sleep outcomes in the ICU: Reflections on the DREAM trial

**DOI:** 10.1016/j.ccrj.2026.100180

**Published:** 2026-04-19

**Authors:** Lukasz Szarpak, Urantuya Battsengel, Michal Pruc

**Affiliations:** Henry JN Taub Department of Emergency Medicine, Baylor College of Medicine, Houston, TX, United States; Institute of Medical Science, The John Paul II Catholic University of Lublin, Lublin, Poland; Department of Medical Informatics and Telemedicine, Medical University of Warsaw, Poland; Institute of Medical Science, The John Paul II Catholic University of Lublin, Lublin, Poland

**Keywords:** DREAM, Temazepam ICU Sleep

To the Editor,

We read with great interest the recent trial by Showler et al. evaluating temazepam for sleep promotion in critically ill patients.^1^ The study addresses a clinically important question since disturbed sleep is common in the intensive care unit (ICU) and remains difficult to manage effectively.^2^^,^^3^ We commend the authors for conducting a randomised, blinded, placebo-controlled trial in an area where practice often outpaces evidence. However, the findings should be interpreted with caution because the study’s central methodological limitation lies in its primary outcome assessment. The total sleep time was estimated by structured hourly nurse observation rather than by a validated physiological measure of sleep. In critically ill patients, this distinction is not trivial. Sedation, hypoactivity, delirium, and altered consciousness may all resemble sleep at the bedside while differing substantially from restorative sleep on electrophysiological assessment.^2^^,^^4^ As a result, the reported increase in nurse-observed total sleep time may not necessarily represent a true improvement in sleep itself.

This concern is reinforced by the discordance between the apparent signal in observed sleep duration and the absence of improvement in patient-reported sleep quality.^1^ If temazepam had produced a clinically meaningful enhancement in sleep, one might expect at least some parallel improvement in the patient experience of sleep. The lack of such a finding raises the possibility that the intervention increased the appearance of sleep more than its quality or restorative value. In that sense, the study may be at risk of overestimating benefit because its primary endpoint captures observed quiescence rather than validated sleep architecture.

The trial design also limits the strength of causal inference. As a small, single-centre pilot study with 56 evaluable patients, it was inherently vulnerable to imprecision and baseline imbalance. Moreover, enrolment depended on weekday staff availability and on the treating clinician’s judgement that a pharmacological sleep aid was indicated, which may have introduced selection effects and reduced generalisability.^1^ The retrospective trial registration after enrolment had begun, while acknowledged transparently by the authors also warrants mention when interpreting an exploratory efficacy signal.

These issues are especially important in the context of benzodiazepine use in the ICU. Even if temazepam were to modestly increase observed nocturnal sleep time, that benefit would need to be weighed against longstanding concerns regarding delirium and other adverse neuropsychiatric effects associated with this drug class in critically ill patients.^3^^,^^5^

This trial contributes useful preliminary data, but it does not yet establish that temazepam meaningfully improves sleep in the ICU. Larger multicentre studies using validated objective sleep measures and patient-centred outcomes will be needed before these findings can support routine clinical use.

## CRediT authorship contribution statement

Conceptualisation: L.S., M.M., and M.P. Writing - original draft: L.S., M.M., and M.P.; Writing - review & editing: L.S., M.M., and M.P.

## Funding

This research did not receive any specific grant from funding agencies in the public, commercial, or not-for-profit sectors.

## Conflict of interest

The authors declare that they have no known competing financial interests or personal relationships that could have appeared to influence the work reported in this paper.
